# Effect of Quadrature Control Mode on ZRO Drift of MEMS Gyroscope and Online Compensation Method

**DOI:** 10.3390/mi13030419

**Published:** 2022-03-08

**Authors:** Feng Bu, Shuwen Guo, Bo Fan, Yiwang Wang

**Affiliations:** 1 School of Electronic and Information Engineering, Suzhou Vocational University, Suzhou 215104, China; bf_suda@126.com (F.B.); wyiwang@163.com (Y.W.); 2 School of Electronic and Information Engineering, Soochow University, Suzhou 215006, China; 3 School of Electrical Engineering, University of South China, Hengyang 421001, China; fanbohysd@163.com

**Keywords:** MEMS gyroscope, closed-loop detection, quadrature control, ZRO drift, online compensation

## Abstract

The quadrature coupling error is an important factor that affects the detection output of microelectromechanical system (MEMS) gyroscopes. In this study, two quadrature error control methods, quadrature force-to-rebalance control (Mode I) and quadrature stiffness control (Mode II) were analyzed. We obtained the main factors affecting the zero-rate output (ZRO) under force-to-rebalance (FTR) closed-loop detection. The analysis results showed that the circuit phase delay in Mode I caused the quadrature channel to leak into the in-phase channel. However, in Mode II, the quadrature coupling stiffness was corrected in real time, which effectively improved the stability of the ZRO. The changes in the vibration displacement and Q-factor were the main factors for the ZRO drift in Mode II. Therefore, we propose an online compensation method for ZRO drift based on multiparameter fusion. The experimental results on a cobweb-like disk resonator gyroscope (CDRG) with a 340 k Q-factor showed that the bias instability (BI) of Mode II was significantly better than that of Mode I. After online compensation, the BI reached 0.23°/h, and the bias repeatability reached 3.15°/h at room temperature.

## 1. Introduction

A microelectromechanical system (MEMS) vibrating gyroscope based on the Coriolis effect has many advantages, such as its small size, light weight, and low cost [1,2]. It has wide application prospects in the military and civil engineering fields. The axisymmetric structure helps improve the energy transfer efficiency and vibration resistance, and has become an important candidate for high-performance MEMS gyroscopes. Compared to open-loop detection, force-to-rebalance (FTR) closed-loop detection can extend bandwidth, increase range, and improve detection stability [2,3,4].

Due to imperfections in micro-processing technology, the uniformity of the structure is difficult to control [5], which inevitably leads to damping coupling and stiffness coupling interference, resulting in in-phase and quadrature errors. The quadrature error was 90° out of phase with the Coriolis force. Ideally, quadrature errors can be eliminated by 90° demodulation. However, the gyroscope control circuit generates phase delays, which cause the drive mode to operate in a non-resonant state. This phase delay directly affects the demodulation accuracy, resulting in interference of the quadrature error with the zero-rate output (ZRO). There are two main quadrature error control methods under FTR closed-loop detection: quadrature FTR control (Mode I) and quadrature stiffness control (Mode II). In Mode I, a feedback force with the same frequency and phase as the quadrature force was applied to the sense mode to counteract the effect of the quadrature force. In Mode II, the quadrature coupling stiffness was adjusted by applying a DC voltage to the stiffness-axis tuning electrode on the gyroscope to inhibit the formation of the quadrature force.

Mode I has been used in [6,7,8,9,10,11,12]. Among them, a bias instability (BI) of 4°/h was realized on a tuning fork gyroscope with a Q-factor of 2 k [6]. A BI of 0.9°/h was realized on a quadrupole mass gyroscope (QMG) with a Q-factor of 1.2 million [7]. A BI of 3.0°/h was realized on a pendulum gyroscope with a Q-factor of 1 k [8]. A BI of 1.5°/h was realized in a three-fold symmetric gyroscope with a Q-factor of 18 k. A BI of 2.8°/h was realized on a triangular-electrode-based gyroscope with a Q-factor of 7 k [10]. A BI of 1.5°/h was realized on a ring gyroscope with a Q-factor of 22 k, along with mode-matching technology [11]. A BI of 0.2°/h was realized on a ladder gyroscope with a Q-factor of 120 k [12].

Mode II was used in [13,14,15,16,17]. Among them, a BI of 0.83°/h was realized on a gyroscope with a 9 k Q-factor combined with mode-matching technology [13]. A BI of 0.18°/h was realized on a disk gyroscope with a 100 k Q-factor [14]. A BI of 0.015°/h was realized on a honeycomb disk resonator gyroscope (HDRG) with a 650 k Q-factor [15]. A BI of 0.09°/h was realized on a slot-structure gyroscope with a 26 k Q-factor by combining a constant frequency drive and mode-matching [16]. A BI of 0.01°/h was realized on a birdbath resonator gyroscope (BRG) with a 1.5 million Q-factor [17]. In contrast, most high-performance gyroscopes reported in recent years have adopted Mode II.

Based on previous work on gyroscope closed-loop detection [18,19,20], this study compares and analyzes the two quadrature error control modes (Mode I and Mode II) under closed-loop detection. System models of the two control modes were constructed, and the effects of circuit phase delay, quadrature coupling, in-phase coupling, phase of drive, and sense mode on the ZRO were analyzed. Moreover, an online ZRO bias compensation method based on multiparameter fusion is proposed. Comparative experiments were performed on a cobweb-like disk resonator gyroscope (CDRG) [21,22]. The results showed that Mode II is more suitable for high-Q gyroscopes whose quadrature error fluctuates easily, and the bias stability can be effectively improved by compensation.

## 2. Gyroscope Dynamic Model with Structural Error

The vibratory gyroscope model can be described using a second-order mass-damper-spring system. Manufacturing process errors create stiffness and damping asymmetries, such that the main stiffness axis and main damping axis have stiffness axis deflection angle $\theta_{\omega }$ and damping axis deflection angle $\theta_{\tau }$ with the reference coordinate system x-o-y. In addition, there may be a deflection angle between the direction of the excitation force input electrode and the reference coordinate system. We set $\alpha_{x}$ as the driving force deflection angle in drive mode and $\alpha_{y}$ as the feedback force deflection angle in the sense mode. The establishment process of the dynamic model has been described in detail in the relevant literature [23,24], and is described by

(1) $$\begin{array}{l} \left[\begin{array}{c} \ddot{x}\\ \ddot{y} \end{array}\right]+\left[\begin{array}{cc} \frac{2}{\tau }+\Delta \left(1/\tau \right)\cos2\theta_{\tau } & \Delta \left(1/\tau \right)\sin2\theta_{\tau }\\ \Delta \left(1/\tau \right)\sin2\theta_{\tau } & \frac{2}{\tau }-\Delta \left(1/\tau \right)\cos2\theta_{\tau } \end{array}\right]\left[\begin{array}{c} \dot{x}\\ \dot{y} \end{array}\right]+\left[\begin{array}{cc} \omega^{2}+\omega \Delta \omega \cos2\theta_{\omega } & \omega \Delta \omega \sin2\theta_{\omega }\\ \omega \Delta \omega \sin2\theta_{\omega } & \omega^{2}-\omega \Delta \omega \cos2\theta_{\omega } \end{array}\right]\left[\begin{array}{c} x\\ y \end{array}\right]\\ \;\;\;\;\;\;\;\;\;\;=\frac{1}{m}\left[\begin{array}{cc} \cos\alpha_{x} & -\sin\alpha_{y}\\ \sin\alpha_{x} & \cos\alpha_{y} \end{array}\right]\left[\begin{array}{c} F_{x}\\ F_{y} \end{array}\right]+2nA_{g}\Omega \left[\begin{array}{c} \dot{y}\\ -\dot{x} \end{array}\right] \end{array}$$

Here,

(2) $$\frac{2}{\tau }=\frac{1}{\tau_{x}}+\frac{1}{\tau_{y}},\;\Delta \left(1/\tau \right)=\frac{1}{\tau_{x}}-\frac{1}{\tau_{y}},\;\omega \Delta \omega =\frac{\omega_{x}^{2}-\omega_{y}^{2}}{2},\;\omega^{2}=\frac{\omega_{x}^{2}+\omega_{y}^{2}}{2}$$

where x and y are the displacement of the gyroscope oscillator in drive mode and sense mode, respectively; τ is the attenuation time constant $\tau_{x,y}=2Q_{x,y}/\omega_{x,y}$; $\omega_{x,y}$ is the resonant frequency of drive or sense mode; $Q_{x,y}$ is the quality factor of drive or sense mode; m is the mass; $F_{x}$ and $F_{y}$ are the excitation forces of the drive and sense modes, respectively; $A_{g}$ is the angular gain; n is the mode order; Ω is the input rotation rate; and $A_{x}$ is the amplitude of displacement x.

Because $\alpha_{x}$ and $\alpha_{y}$ are usually significantly small, we ignored their impact. The damping coupling coefficient is $c_{yx}=\Delta \left(1/\tau \right)\sin2\theta_{\tau }m$, and the stiffness coupling coefficient is $k_{yx}=\omega \Delta \omega \sin2\theta_{\omega }m$. Then, the amplitude $A_{I}$ of the in-phase coupling force $F_{I}$ and the amplitude $A_{q}$ of the quadrature coupling force $F_{q}$ are expressed as follows:

(3) $$\{\begin{array}{l} A_{I}=-\Delta \left(1/\tau \right)\sin2\theta_{\tau }m\omega_{x}A_{x}\\ A_{q}=-\omega \Delta \omega \sin2\theta_{\omega }mA_{x} \end{array}$$

The higher and more matched the Q-factor of the two modes, the more beneficial it is to reduce $A_{I}$. $A_{q}$ is negatively correlated with the frequency split between the two modes.

## 3. Closed-Loop Control of Drive Mode

**Figure 1:**
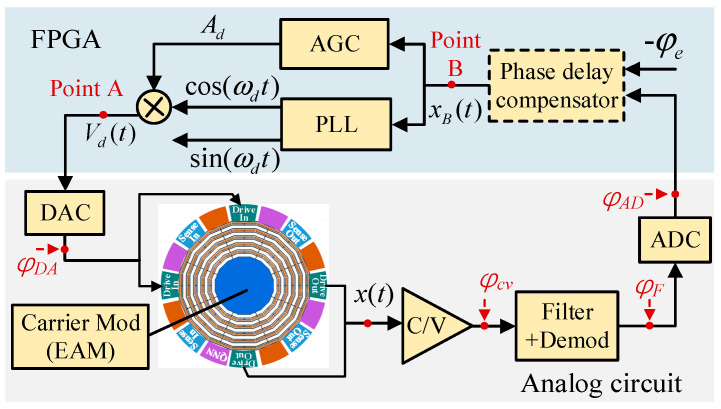
The control scheme of the drive mode.

The drive mode control system is composed of a classical amplitude gain control (AGC) and phase-locked loop (PLL) system, as shown in Figure 1. AGC is used to maintain the vibration displacement amplitude $A_{x}$ at a constant, and the PLL maintains the drive mode in the resonant state ($\omega_{d}=\omega_{x}$). In addition, using the electromechanical amplitude modulation (EAM) signal pickoff method, a high-frequency carrier is applied to the gyroscope mass block to modulate the amplitude of the vibration displacement signal to suppress the feed-through interference.

Because the analog circuits of the drive and sense modes are completely consistent, the phase delay of each part is the same. Let $\varphi_{CV}$ be the phase delay of the C/V circuit; $\varphi_{F}$ be the phase delay of the demodulation and filter circuit; and $\varphi_{DA}$ and $\varphi_{AD}$ be the phase delays of the DAC and ADC circuits, respectively. The total phase delay of the analog circuit is then given by $\varphi_{e}=\varphi_{CV}+\varphi_{F}+\varphi_{AD}+\varphi_{DA}$.

Let the drive mode excitation signal $V_{d}(t)$ generated by the FPGA be (point A) given by

(4) $$V_{d}(t)=A_{d}\cos(\omega_{d}t)$$

where $A_{d}$ is the signal amplitude and $\omega_{d}$ is the signal frequency. The excitation signal is loaded on the gyroscope electrode after the DAC, and the resulting vibration displacement x(t) of the drive mode is

(5) $$x(t)=A_{x}\cos\left(\omega_{d}t+\varphi_{DA}+\varphi_{x}\right)$$

where $\varphi_{x}$ is the drive-mode phase. Due to the existence of the circuit phase delay $\varphi_{e}$, when there is no phase delay compensation, the vibration displacement signal (point B) entering the PLL is $x_{B}(t)=k_{e}A_{x}\cos\left(\omega_{d}t+\varphi_{e}+\varphi_{x}\right)$. The function of the PLL is to lock the phase difference between the input signal $x_{B}(t)$ and the output signal $\cos(\omega_{d}t)$ at $-90^{o}$:

(6) $$\varphi_{e}+\varphi_{x}=-90^{o}$$

Therefore, in practical applications, it is necessary to compensate for the phase delay of the circuit (i.e., make $\varphi_{e}=0$) to ensure that the driving mode works in the resonant state ($\varphi_{x}=-90^{o}$).

## 4. Closed-Loop Control of the Sense Mode

For gyroscopes with a high Q-factor, the change in the sense mode gain under open-loop detection significantly affects detection stability. In contrast, FTR closed-loop detection can make the detection output insensitive to the sense mode gain, and alleviate the influence of environmental parameters on ZRO and the scale factor [8]. In closed-loop detection, a feedback force was used to offset the Coriolis force. In addition, a quadrature control loop was required to suppress the quadrature displacement on the sense axis to keep the sense mode relatively stationary.

In quadrature control Mode I, a suppression force signal in phase with the drive mode displacement signal *x* was applied to the excitation electrode of the sense mode; hence, the quadrature displacement was suppressed. In quadrature control Mode II, according to the quadrature displacement of the sense mode, the regulating voltage was applied to the stiffness axis tuning electrode to adjust the stiffness axis deflection angle $\theta_{\omega }$ to zero.

### 4.1. Quadrature FTR Control (Mode I)

**Figure 2:**
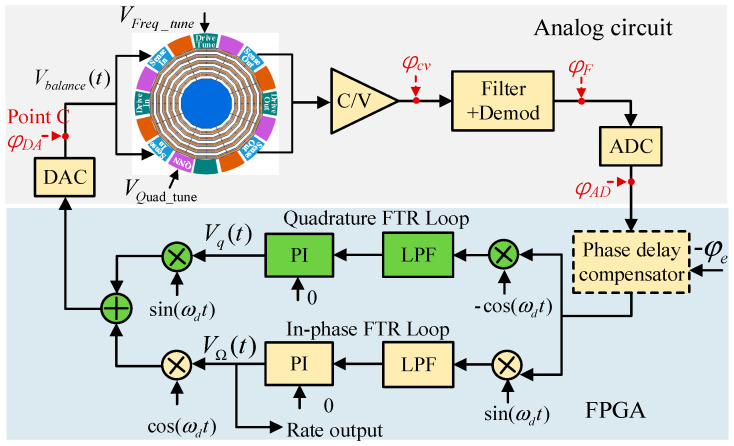
The control scheme of Mode I closed-loop gyroscope system.

The Mode I closed-loop gyroscope system including the quadrature FTR loop and in-phase FTR loop is shown in Figure 2. In a field-programmable gate array (FPGA) digital circuit system, the output of the pickoff circuit was demodulated in the phase and quadrature. After the four-order Butterworth low-pass filter (LPF) with a cut-off frequency of 800 Hz, the magnitude of the Coriolis and quadrature responses were obtained. Thereafter, two feedback forces were generated by the proportional-integral (PI) controller to suppress the Coriolis and quadrature forces and to maintain the sense mode relatively static. The PI output of the in-phase channel was the rotation-rate detection output.

Because the analog circuit of the sense-mode system was consistent with the drive mode, the total circuit phase delay was also $\varphi_{e}$. The above shows that $\varphi_{e}$ caused the drive mode to operate in a nonresonant state ($\varphi_{x}\neq -90^{o}$). Under Mode I control, this led to mutual leakage between the quadrature feedback channel and in-phase feedback channel [18].

According to the vibration displacement x(t) of the drive mode, the in-phase force $F_{\Omega +I}(t)$ (Coriolis force and in-phase coupling force) and quadrature force $F_{q}(t)$ input to the sense mode are expressed as

(7) $$\left\{ \begin{array}{l} F_{\Omega +I}(t)=\left(A_{\Omega }+A_{I}\right)\sin(\omega_{d}t+\varphi_{DA}+\varphi_{x})\\ F_{q}(t)=A_{q}\cos(\omega_{d}t+\varphi_{DA}+\varphi_{x}) \end{array}\right.$$

where $A_{\Omega }$ represents the amplitudes of the Coriolis force, i.e., $A_{\Omega }=-2nA_{g}\Omega m\omega_{x}A_{x}$.

Two digital feedback signals were generated and added through the double-loop FTR system, and an analog feedback signal (point C) was formed after passing through the DAC:

(8) $$V_{balance}(t)=V_{\Omega }\cos(\omega_{d}t+\varphi_{DA})+V_{q}\sin(\omega_{d}t+\varphi_{DA})$$

where $V_{\Omega }$ and $V_{q}$ are the amplitudes of in-phase and quadrature feedback signals, respectively.

According to the principle of FTR closed-loop detection, the sense mode was stationary in the steady state. At this time, the resultant force input to the sense mode was zero:

(9) $$F_{\Omega +I}(t)+F_{q}(t)-V_{balance}(t)K_{vf}=0$$

Substituting Equations (7)–(9), we obtain $V_{\Omega }$ and $V_{q}$ as

(10) $$\left\{ \begin{array}{l} V_{\Omega }=\left[\left(A_{\Omega }+A_{I}\right)\sin(\varphi_{x})+A_{q}\cos(\varphi_{x})\right]/K_{vf}\\ V_{q}=\left[\left(A_{\Omega }+A_{I}\right)\cos(\varphi_{x})-A_{q}\sin(\varphi_{x})\right]/K_{vf} \end{array}\right.$$

ZRO is the output under a zero-rate input, that is, ${V_{\Omega }|}_{A_{\Omega }=0}$. Then, the ZRO in Mode I is expressed as:

(11) $${\mathrm{ZRO}}_{1}=\left[A_{I}\sin(\varphi_{x})+A_{q}\cos(\varphi_{x})\right]/K_{vf}$$

### 4.2. Quadrature Stiffness Control (Mode II)

**Figure 3:**
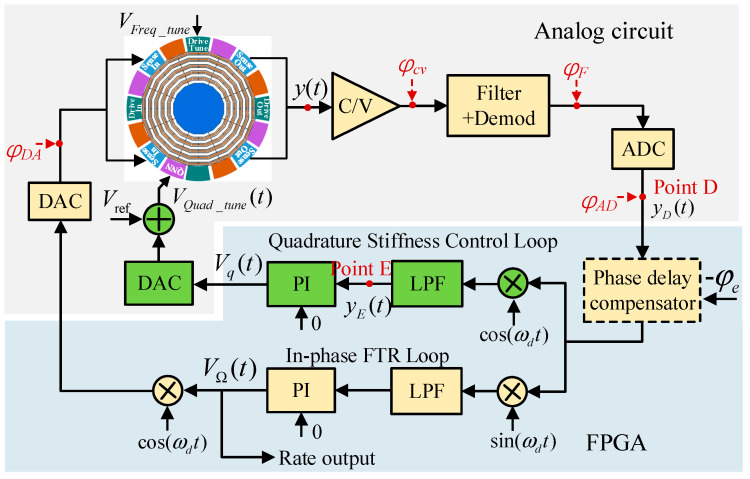
The control scheme of the Mode II closed-loop gyroscope system.

The Mode II closed-loop gyroscope system, including the quadrature stiffness control loop and the in-phase FTR loop, is shown in Figure 3. The output of the quadrature PI controller was directly converted into a voltage signal through the DAC, added with a bias voltage $V_{ref}$, and loaded on the stiffness axis tuning electrode for quadrature coupling stiffness correction.

The sense modal displacement y(t) caused by the in-phase and quadrature forces is given by the following:

(12) $$y(t)=A_{ys}\left[\left(A_{\Omega }+A_{I}\right)\sin(\omega_{d}t+\varphi_{DA}+\varphi_{x}+\varphi_{y})+A_{q}\cos(\omega_{d}t+\varphi_{DA}+\varphi_{x}+\varphi_{y})\right]$$

where $A_{ys}$ and $\varphi_{y}$ are the mechanical gain and the phase of the sense mode, respectively. When the frequencies of the two modes match ($\omega_{y}=\omega_{x}$), $A_{ys}$ becomes the largest and $\varphi_{y}=-90^{o}$.

After passing through the signal pickoff circuit and ADC, signal y(t) becomes (point D).

(13) $$\begin{array}{l} y_{D}(t)=K_{e}A_{ys}\left[\left(A_{\Omega }+A_{I}\right)\sin(\omega_{d}t+\varphi_{e}+\varphi_{x}+\varphi_{y})+A_{q}\cos(\omega_{d}t+\varphi_{e}+\varphi_{x}+\varphi_{y})\right]\\ \;\;\;\;\overset{\varphi_{e}+\varphi_{x}=-90^{o}}{=}K_{e}A_{ys}\left[-\left(A_{\Omega }+A_{I}\right)\cos(\omega_{d}t+\varphi_{y})+A_{q}\sin(\omega_{d}t+\varphi_{y})\right] \end{array}$$

When there is no phase delay compensation, after being demodulated by $\cos(\omega_{d}t)$ and LPF, the signal $y_{D}(t)$ becomes (point E)

(14) $$y_{E}(t)=\frac{1}{2}K_{e}A_{ys}\left[-\left(A_{\Omega }+A_{I}\right)\cos(\varphi_{y})+A_{q}\sin(\varphi_{y})\right]$$

In the quadrature stiffness control loop, the PI controller always keeps $y_{E}(t)=0$ by adjusting the quadrature correction voltage. Hence,

(15) $$A_{q}=\left(A_{\Omega }+A_{I}\right)\cot(\varphi_{y})$$

According to the above analysis, $V_{\Omega }$ in the in-phase FTR loop is given by

(16) $$V_{\Omega }=\left[\left(A_{\Omega }+A_{I}\right)\sin(\varphi_{x})+A_{q}\cos(\varphi_{x})\right]/K_{vf}$$

Substituting Equation (13) into (16), we obtain

(17) $$V_{\Omega }=\left(A_{\Omega }+A_{I}\right)\left[\sin(\varphi_{x})+\cot(\varphi_{y})\cos(\varphi_{x})\right]/K_{vf}$$

Then, ZRO in Mode II is expressed as

(18) $${\mathrm{ZRO}}_{2}=A_{I}\left[\sin(\varphi_{x})+\cot(\varphi_{y})\cos(\varphi_{x})\right]/K_{vf}$$

### 4.3. Comparative Analysis

We assume that the frequency split Δf is 1 Hz and the angles $\theta_{\omega }$ and $\theta_{\tau }$ are 0.1° and 1°, respectively. When the drive mode works in the resonant state ($\omega_{x}=\omega_{d}$), $A_{I}$ and $A_{q}$ can be estimated (see Table 1) using Equation (3), and the parameters of the CDRG gyroscope are presented in Table 2. For high-Q-factor gyroscopes, $A_{q}$ is usually several orders of magnitude larger than $A_{I}$, without perfect quadrature stiffness correction.

Equation (11) shows that ${\mathrm{ZRO}}_{1}$ is primarily affected by $A_{q}$ and $\varphi_{x}$. The main function of circuit phase delay $\varphi_{e}$ is to cause $\varphi_{x}\neq -90^{o}$, which introduces the quadrature interference term $A_{q}\cos(\varphi_{x})$ to ${\mathrm{ZRO}}_{1}$. In a high-Q gyroscope $A_{q}(t)>>A_{I}(t)$; hence, $A_{q}$ is easy to change under the influence of temperature. The constant term in $A_{q}$ is equivalent to introducing a fixed bias to ${\mathrm{ZRO}}_{1}$, while the varying term is equivalent to introducing an uncontrollable low-frequency fluctuation, which leads to a large drift in ${\mathrm{ZRO}}_{1}$. Even if $A_{q}$ is corrected to approximately 0 by a fixed correction voltage in the start-up stage, the changes in ambient temperature and other factors will lead to fluctuations in $A_{q}$ and $\varphi_{e}$, resulting in a large drift of ${\mathrm{ZRO}}_{1}$.

Equation (18) shows that ${\mathrm{ZRO}}_{2}$ is no longer affected by $A_{q}$, so the bias value and bias stability will be effectively improved. ${\mathrm{ZRO}}_{2}$ is affected by $\varphi_{y}$. When $\varphi_{x}\neq -90^{o}$ and $\varphi_{y}\neq -90^{o}$, the interference term $\cot(\varphi_{y})\cos(\varphi_{x})$ is introduced into ZRO. After a one-time circuit phase delay compensation, $\varphi_{x}$ will be close to $-90^{o}$; hence, $\left|\sin(\varphi_{x})\right|>>\left|\cos(\varphi_{x})\right|$. In addition, closed-loop control keeps the sense mode relatively stationary, effectively suppresses the influence of frequency splitting, and expands the mechanical bandwidth [20,25]. In other words, when the frequency difference between the two modes is less than the mechanical bandwidth, the sense mode is approximately in the mode-matched state, that is, $\varphi_{y}\approx -90^{o}$. Therefore, the influence of the interference term $\cot(\varphi_{y})\cos(\varphi_{x})$ was significantly small.

To intuitively express the influence of various factors in Mode I and Mode II on ZRO, it is assumed that $\theta_{\tau }$ and Δf are fixed at 1° and 1 Hz, respectively. $\theta_{\omega }$ can be changed by correction (i.e., $A_{q}$ is variable), and the phase $\varphi_{x}$ changes from −50° to −130°.

**Figure 4:**
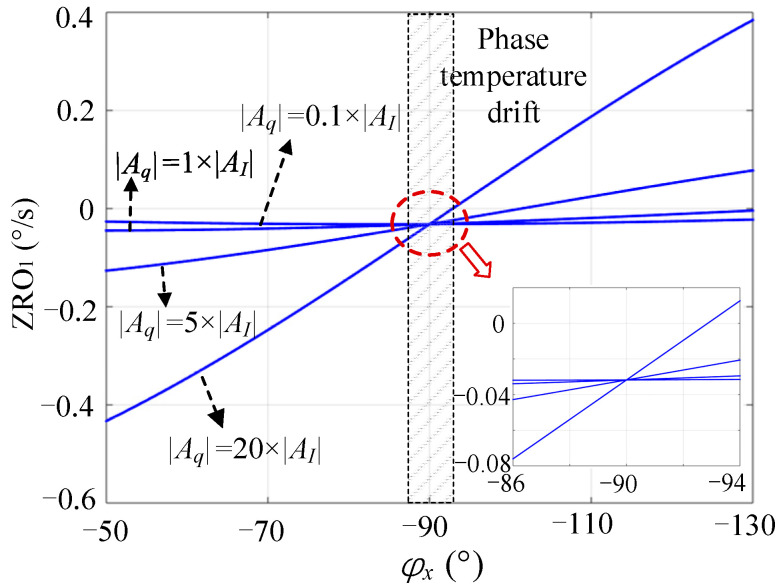
Effect of $\varphi_{x}$ and $A_{q}$ on ${\mathrm{ZRO}}_{1}$.

The calculated values of ${\mathrm{ZRO}}_{1}$ under different $\varphi_{x}$ and $A_{q}$ are shown in Figure 4; ${\mathrm{ZRO}}_{1}$ changes greatly under different $\varphi_{x}$ and the change increases with $A_{q}$; ${\mathrm{ZRO}}_{1}$ is relatively stable only when $A_{q}$ is corrected to a significantly small value. However, in practical applications, $A_{q}$ changes as the environmental factors change. Even if $A_{q}$ is corrected to zero when the gyroscope system is started, its subsequent long-term stability cannot be guaranteed.

**Figure 5:**
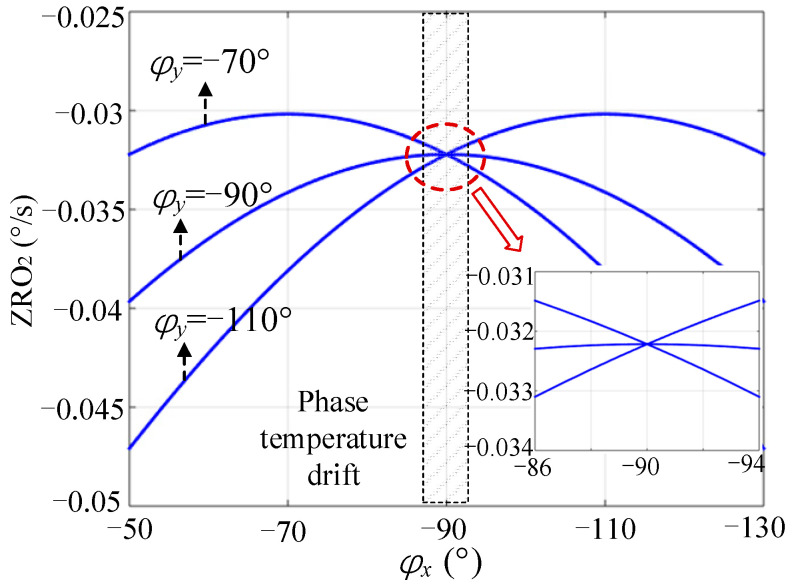
Effect of $\varphi_{x}$ and $\varphi_{y}$ on ${\mathrm{ZRO}}_{2}$.

Under the same conditions, the calculated values of ${\mathrm{ZRO}}_{2}$ under different $\varphi_{x}$ and $\varphi_{y}$ are shown in Figure 5. The comparison shows that the change in ${\mathrm{ZRO}}_{2}$ was significantly less than that of ${\mathrm{ZRO}}_{1}$. In practical applications, $\varphi_{e}$ may change within ±5 degrees in the temperature range of commercial grade [18,26], which will cause fluctuations in $\varphi_{x}$ near $-90^{o}$. In this case, the stability of ${\mathrm{ZRO}}_{2}$ was significantly better than that of ${\mathrm{ZRO}}_{1}$. In addition, ${\mathrm{ZRO}}_{2}$ was also affected by $\varphi_{y}$; however, the change was marginal. In the mode-matched state ($\varphi_{y}=-90^{o}$), ${\mathrm{ZRO}}_{2}$ was more stable.

In summary, in addition to the in-phase error component in ZRO under the control of Mode I, the shifting of $\varphi_{x}$ introduced a large quadrature error. Under the control of Mode II, ZRO was affected only by the in-phase error. For high-Q gyroscopes, the quadrature error was much greater than the in-phase error; thus, Mode II was more suitable for high-Q gyroscopes, which could effectively improve ZRO stability.

## 5. Online Compensation for ZRO Drift under Mode II

Although Mode II had better ZRO stability than Mode I, there was still obvious drift due to the rise in chip temperature during the power-on stage. Therefore, it was necessary to analyze and compensate for the main factors leading to drift in Mode II. According to Equation (3), the in-phase error $A_{I}$ is related to the vibration displacement amplitude $A_{x}$. Because displacement x(t) is excited by the driving signal $V_{d}(t)$, we have

(19) $$A_{x}=A_{d}K_{vf}A_{xs}$$

where $A_{xs}$ denotes the mechanical gain of the drive mode. In the drive mode resonance state (i.e., $\omega_{d}=\omega_{x}$),

(20) $$A_{xs}=\frac{1}{\omega_{x}^{2}m\sqrt{{\left(1-{\left(\omega_{d}/\omega_{x}\right)}^{2}\right)}^{2}+{\left(\omega_{d}/\omega_{x}Q_{x}\right)}^{2}}}\approx \frac{Q_{x}}{\omega_{x}^{2}m}$$

Combined with Equations (3) and (18)–(20), ${\mathrm{ZRO}}_{2}$ can be approximately expressed as follows

(21) $$\begin{array}{l} {\mathrm{ZRO}}_{2}\approx A_{I}\sin(\varphi_{x})/K_{vf}\\ \;\;\;\;\;\;\;\;\;\;\;\approx -\Delta \left(1/\tau \right)\sin2\theta_{\tau }m\omega_{x}A_{x}\sin(\varphi_{x})/K_{vf}\\ \;\;\;\;\;\;\;\;\;\;\;\approx -\Delta \left(1/\tau \right)\sin2\theta_{\tau }A_{d}\frac{Q_{x}}{\omega_{x}}\sin(\varphi_{x}) \end{array}$$

Next, we analyzed the main factors leading to ZRO temperature drift. As can be seen from Equation (21), ${\mathrm{ZRO}}_{2}$ is affected by Δ(1/τ), $\theta_{\tau }$, $A_{d}$, $Q_{y}$, $\omega_{x}$, and $\varphi_{x}$. Because the damping axis deflection angle $\theta_{\tau }$ cannot be directly observed or estimated, it was not considered here. During the gyroscope power-on stage (the chip temperature rises from approximately 20 °C to 32 °C), we measured the changes in other relevant factors, as shown in Table 2. Among these, $\omega_{x}$ and $A_{d}$ could be observed online from the control system, and $\varphi_{x}$ was measured using the method in [18]. $Q_{x}$ and $Q_{y}$ were obtained through an offline free attenuation vibration test, and $\omega_{y}$ was obtained through an offline frequency sweep for calculating Δ(1/τ).

Table 2 shows that the changes in $\omega_{x}$ and $\varphi_{x}$ were very small and had a limited effect on the ZRO drift. For Δ(1/τ), because CDRG had a symmetrical structure, the change trajectories of the frequency and Q-factor of the two modes with temperature were essentially the same [22]; therefore, the change in Δ(1/τ) was not significant. In contrast, the changes in $A_{d}$ and $Q_{x}$ were large, which contributed significantly to the ZRO drift. Among them, the change in $A_{d}$ was mainly caused by the change in the vibration displacement $A_{x}$ and circuit gain.

According to the above analysis, to realize the online compensation of ZRO, it was necessary to monitor the two main influencing factors, $A_{d}$ and $Q_{x}$, in real time. However, $Q_{x}$ could not be monitored directly. Fortunately, the resonant frequency could be used to estimate the change in Q-factor. According to a previous study [22], the temperature coefficient of the Q-factor (TCQ) was taken as the exponent of the temperature *T*, that is, $Q_{x}\propto T^{-TCQ}$. For CDRG, TCQ was approximately 2. In addition, the frequency was approximately linear with the temperature *T*, that is, $\omega_{x}=\omega_{x0}(1+k_{\omega }\Delta T)$. Therefore, we used $\omega_{x}$ to estimate the change of $Q_{x}$.

**Figure 6:**
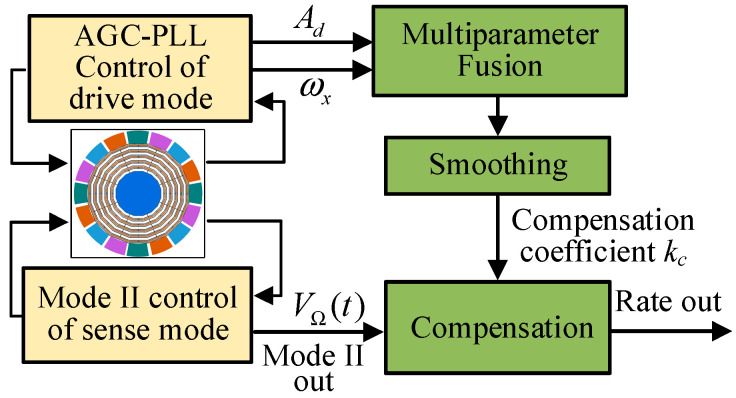
ZRO online compensation method.

The proposed ZRO online compensation method based on multiparameter fusion is illustrated in Figure 6. In the multiparameter fusion module, the compensation parameter $k_{c}$ was calculated according to the real-time change of each parameter relative to the initial value during the power-on stage. Because the temperature drift was a slow changing process, to reduce the interference of noise and outliers, $k_{c}$ was smoothed and then used to compensate for the temperature drift of ZRO. The calculation expression for $k_{c}$ is

(22) $$k_{c}=1-\Delta A_{d}(t)/A_{d0}+\Delta Q_{x}(t)/Q_{x0}+\Delta (1/\omega_{x}(t))/\left(1/\omega_{x0}\right)$$

where $A_{d0}$, $Q_{x0}$ and $\omega_{x0}$ are the initial values during the power-on stage. $\Delta A_{d}(t)$, $\Delta Q_{x}(t)$ and $\Delta (1/\omega_{x}(t))$ are the difference between the real-time monitoring value and the initial value.

## 6. Experimental Results

### 6.1. Gyroscope and Control Circuit

**Figure 7:**
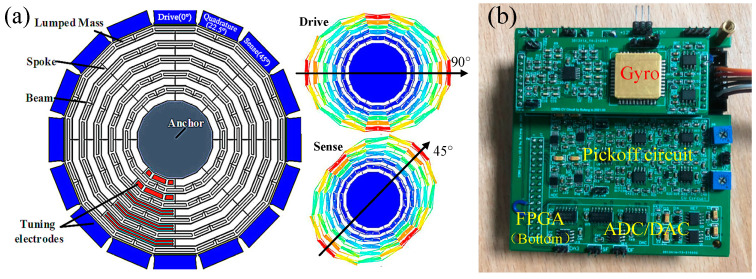
(**a**) Structure of the CDRG and the (**b**) control circuit.

A vacuum-packed CDRG designed by Soochow University was used in this experiment [21,22]. Instead of the traditional ring structure, CDRG uses the latest polygon structure, which effectively reduces the structural symmetry error, resulting in a significantly small frequency split. A frequency split of ∆f < 0.05 Hz could be achieved by electrostatic tuning. The drive mode was in the directions of 0° and 90°, and the sense mode was in the directions of 45° and 135°. The Q-factors of the two modes were as high as 340k. The internal structure of the gyroscope and the control circuit are shown in Figure 7.

A Xilinx artix-7 series FPGA was used as the gyroscope digital control system platform. The programming language was Verilog, the input clock frequency was 100 MHz, and the working rate was set to 1.6 × 10^−6^ s. Relevant data were collected through serial ports and LabVIEW at a sampling rate of 5 Hz. Table 3 lists some of the parameters of the gyroscope.

### 6.2. Measurement of Circuit Phase Delay

**Figure 8:**
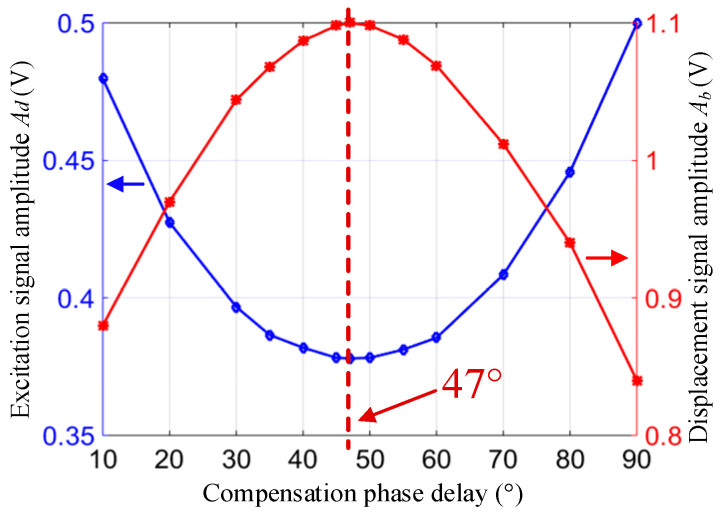
Influence of different compensation phase delays on the excitation signal amplitude.

To observe the actual influence of $\varphi_{x}$ on ZRO, we needed to know the value of the circuit phase delay $\varphi_{e}$, and to then set different compensation phase delay values to achieve different values of $\varphi_{x}$. There are several methods for measuring $\varphi_{e}$, such as (1) a method based on the amplitude of the excitation signal or the amplitude of the vibration response under the drive mode closed-loop and (2) a method based on the coupling relationship between quadrature feedback and in-phase feedback under a double-loop FTR closed-loop [18]. The first method was adopted for visual representation. First, the drive mode closed-loop system adopted the AGC-PLL control scheme and the amplitude $A_{b}$ of the drive mode displacement signal was locked at 2 V. The amplitude $A_{d}$ of the excitation signal under different compensation phase delays was tested. Then, the drive mode only adopted the PLL control, set the $A_{d}$ = 0.2 V, and tested $A_{b}$ under different compensation phase delays, as shown in Figure 8.

This figure shows that when the compensation phase delay was approximately 47°, the amplitude of the excitation signal was the lowest and the vibration displacement amplitude was the largest, indicating that the drive mode worked in the resonant state ($\varphi_{x}\approx -90^{o}$). In other words, the phase delay of the circuit at room temperature was $\varphi_{e}\approx -47^{o}$.

### 6.3. Comparison of Influence $\varphi_{x}$ on ZRO

**Figure 9:**
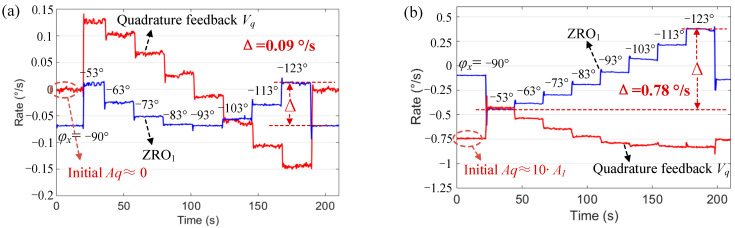
${\mathrm{ZRO}}_{1}$ at different $\varphi_{x}$ in Mode I. (**a**) The initial quadrature error is adjusted to 0 and (**b**) the initial quadrature error is not 0.

**Figure 10:**
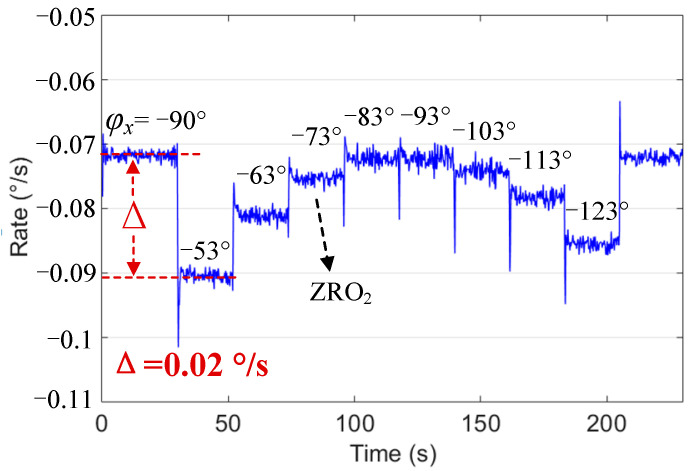
${\mathrm{ZRO}}_{2}$ at different $\varphi_{x}$ in Mode II.

The experiment was used to verify the influence of the drive mode phase $\varphi_{x}$ on ZRO. In the experiment, the compensation phase delay was set to 10°–80° and the change step was 10°, which made $\varphi_{x}$ equal to −53°, −63°, −73°, −83°, −93 °, −103°, −113°, and −123°. The ZRO at different $\varphi_{x}$ in Mode I and Mode II are shown in Figure 9 and Figure 10, respectively. Among them, Mode I was divided into two cases: the initial quadrature force amplitude $A_{q}$ was corrected to 0 and not corrected to 0, which is realized by setting different fixed quadrature tuning voltages $V_{Quad\_tune}$. When $V_{Quad\_tune}$ = 5.6 V, $A_{q}$ was corrected to approximately 0; simultaneously, we set the fixed frequency tuning voltage $V_{Freq\_tune}$ = 10 V.

The quadrature feedback signal amplitude $V_{q}$ in Mode I changed significantly at different $\varphi_{x}$. Even when the initial $A_{q}$ was corrected to 0, the maximum change in ${\mathrm{ZRO}}_{1}$ (Δ) also reached 0.09°/s. This shows that the phase change led to a drastic change in $A_{q}$; hence, the change in ${\mathrm{ZRO}}_{1}$ was also significant. In Mode II, $A_{q}$ was corrected to approximately 0 in real time, and ${\mathrm{ZRO}}_{2}$ was mainly affected by in-phase coupling $A_{I}$; the change was significantly smaller, with a maximum change (Δ) of 0.02°/s. Therefore, when $\varphi_{x}$ drifted around −90° due to changes in ambient temperature and other reasons, the stability of ${\mathrm{ZRO}}_{2}$ became significantly greater than that of ${\mathrm{ZRO}}_{1}$.

In addition, we attempted to change the sense mode phase $\varphi_{y}$ by changing the tuning voltage $V_{Freq\_tune}$ to verify that ${\mathrm{ZRO}}_{2}$ was affected by $\varphi_{y}$. The change in ${\mathrm{ZRO}}_{2}$ was not obvious; in other words, the change in $\varphi_{y}$ was not obvious. This may be because the closed-loop control expanded the mechanical bandwidth of the sense mode. When the frequency split was less than the bandwidth, it could be in the mode-matched state; that is, $\varphi_{y}$ is close to −90°.

### 6.4. Comparison of Bias Stability

**Figure 11:**
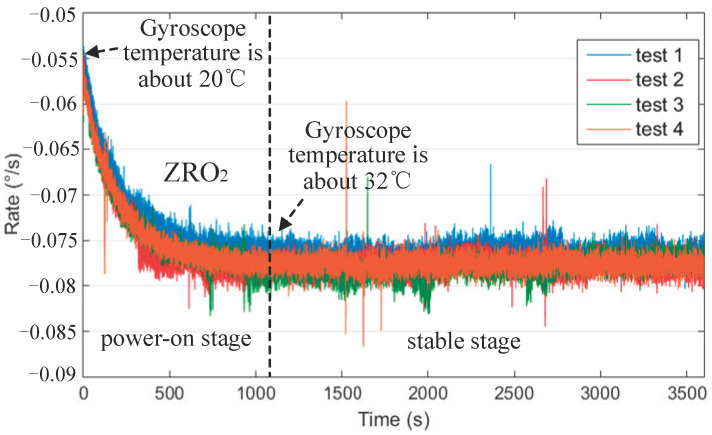
${\mathrm{ZRO}}_{2}$ data of gyroscope cold start at room temperature.

First, we tested the repeatability of ZRO. The cold started the gyroscope system for the ZRO data acquisition at room temperature. The ZRO test was performed four times under each control mode. The sampling time of each test was 1 h, sampling frequency was 5 Hz, and power-off time of each test was 0.5 h. The ${\mathrm{ZRO}}_{2}$ data for the four tests are presented in Figure 11. The first 20 min were the power-on stage, and the last 40 min were the stable stages for the bias stability analysis.

**Figure 12:**
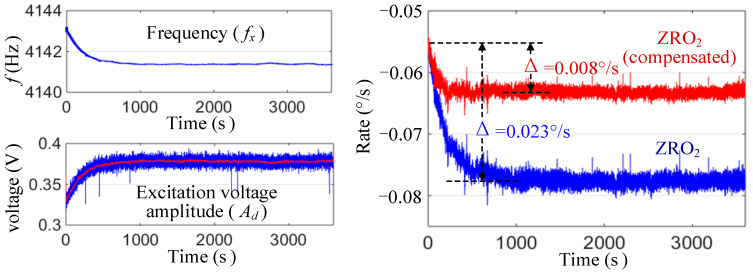
Online compensation process for ${\mathrm{ZRO}}_{2}$ during the power-on stage.

We then tested the effect of the online compensation on the ZRO drift during the power-on stage. Figure 12 shows a comparison of the ${\mathrm{ZRO}}_{2}$ curves before and after the compensation in one test. It can be seen that the ${\mathrm{ZRO}}_{2}$ drift after compensation was significantly reduced, from 0.023°/s to 0.008°/s.

**Figure 13:**
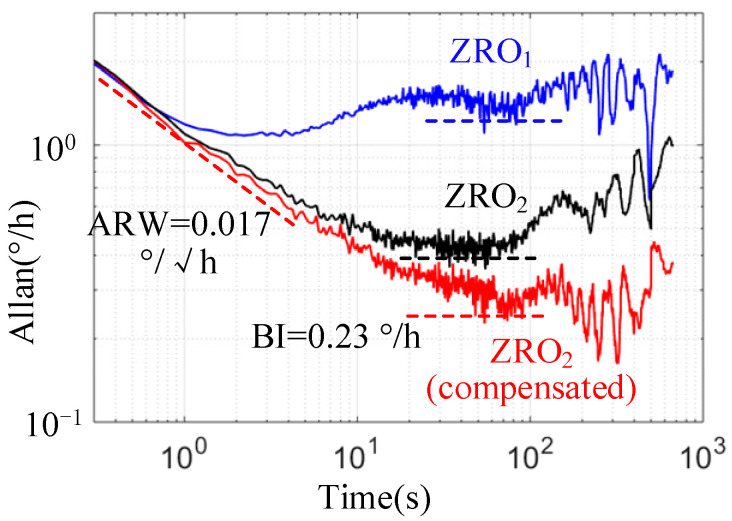
Allan variance curve of ZRO at room temperature.

The Allan variance curves for ${\mathrm{ZRO}}_{1}$, ${\mathrm{ZRO}}_{2}$, and compensated ${\mathrm{ZRO}}_{2}$ during the stable stage (from 1500 s to 3500 s) obtained from one test are shown in Figure 13. The results of the performance comparison are presented in Table 4. The bias repeatability and bias average were calculated based on the results of the four tests, and the initial quadrature coupling $A_{q}$ in Mode I was corrected to approximately 0.

In conclusion, the experimental results show that the BI and bias repeatability of ${\mathrm{ZRO}}_{2}$ reached 0.36°/h and 3.44°/h, respectively, which were approximately four times better than those of ${\mathrm{ZRO}}_{1}$. This was mainly due to the real-time quadrature stiffness control in mode II, which effectively weakened the influence of the quadrature error on ZRO. After online compensation, the BI of ${\mathrm{ZRO}}_{2}$ reached 0.23°/h, and the low frequency band (long-term drift) of Allan variance clearly became smoother, which proved the effectiveness of the multi parameter fusion compensation method.

In addition, the angle random walk (ARW) was basically the same, because the signal processing circuit and in-phase feedback loop in the two modes were completely consistent, so the short-term noise level of ZRO was basically the same.

## 7. Conclusions

In this study, two quadrature error control modes under closed-loop detection of MEMS gyroscopes were compared, and the factors affecting the ZRO were analyzed. Experiments were performed on an axisymmetric gyroscope with a 340 k Q-factor. It was proven that the quadrature stiffness control mode could effectively improve the stability and repeatability of ZRO and make the BI reach 0.36°/h. After online compensation based on multiparameter fusion, BI reached 0.23°/h.

In future work, online compensation technology for a scale factor will be studied to further improve the long-term bias stability.

## Figures and Tables

**Table 1:** Theoretical calculation of the amplitude of the quadrature and in-phase coupling force.

Parameters	$\left|A_{I}\right|(N)$	$\left|A_{q}\right|(N)$
$\theta_{\omega }$ = 1°, $\theta_{\tau }$ = 1°	1.04 × 10^−11^	1.15 × 10^−8^
$\theta_{\omega }$ = 0.1°, $\theta_{\tau }$ = 1°	1.04 × 10^−11^	1.24 × 10^−9^

**Table 2:** Measurement of parameter changes during gyroscope power on stage.

Parameters	Initial Value during Power-On Stage (about 20 °C)	Value during Stable Stage (about 32 °C)	Variation (Δ)	Rate of Change
$\omega_{x}$	4142.9 Hz	4141.6 Hz	1.3 Hz	0.031%
$A_{d}$	0.33 V	0.38 V	0.05 V	−15.15%
$\varphi_{x}$	−88.5°	−89.8°	1.3°	−0.032% ($\sin(\varphi_{x})$)
$Q_{x}$	373.2 k	341.3 k	31.9 k	8.55%
Δ(1/τ) *	1.112 × 10^−3^	1.071 × 10^−3^	0.041 × 10^−3^	3.68%
$\theta_{\tau }$	-	-	-	-

* Calculated using Q-factor and frequency parameters.

**Table 3:** Basic parameters of gyroscope and circuit.

Parameters	Values
Proof mass (m)	1 mg
vibration displacement ($A_{x}$) *	2 μm
Drive-mood resonant frequency ($f_{x}$)	4141.7 Hz
Sense-mood resonant frequency ($f_{y}$)	4140.6 Hz
Drive-mood Q-factor ($Q_{x}$)	341.3 k
Sense-mood Q-factor ($Q_{y}$)	351.5 k
Scale Factor (SF)	147 mV/(°/s)
Carrier signal	6V_pk_@1 MHz

* Estimated by the output voltage amplitude of the drive mode.

**Table 4:** Comparison of bias performance at room temperature.

	Bias Instability (°/h)	ARW (°/√h)	Bias Average (°/s)	Bias Repeatability (°/h)	Bias Drift (°/s)
ZRO_1_ (initial $A_{q}$≈ 0)	1.28	0.020	−0.073	17.01	0.037
ZRO_2_	0.36	0.019	−0.077	3.44	0.023
ZRO_2_ (compensated)	0.23	0.017	−0.063	3.15	0.008
